# Value of ultralong‐term subcutaneous EEG monitoring for treatment decisions in temporal lobe epilepsy: A case report

**DOI:** 10.1002/epi4.12844

**Published:** 2023-10-25

**Authors:** Martin Hirsch, Yulia Novitskaya, Andreas Schulze‐Bonhage

**Affiliations:** ^1^ Epilepsy Center, University Medical Center University of Freiburg Freiburg Germany; ^2^ European Reference Network EpiCare Bron France

**Keywords:** epilepsy treatment, seizure lateralization, subcutaneous EEG, ultralong‐term EEG monitoring

## Abstract

Treatment decisions in epilepsy critically depend on information on the course of the disease, its severity and options for specific local interventions. We here report a patient with pharmaco‐resistant non‐lesional temporal lobe epilepsy with evidence for predominant right temporal epileptogenesis. While seizure frequency had been grossly underestimated for many years, ultralong‐term monitoring with a subcutaneous EEG device revealed actual seizure frequency (66 over 11 months vs four patient‐documented seizures), providing objective data on treatment efficacy and additional supportive lateralizing information that played a decisive role for the choice of surgical treatment, which had been rejected by the patient prior to this information.

## 1 INTRODUCTION

Treatment decisions in epileptology depend crucially on the assessment of patient‐individual burden of disease, considering in particular seizure frequency and severity. So far, the disease evaluation has mostly been based on patient reports on seizure occurrence during a reference period, for example, during the months preceding an outpatient visit at the treating neurologist. However, evidence has been accumulated that reported seizure frequencies are often invalid, as patients underreport even severe seizure types and seizures occurring at night, when they bear the highest SUDEP risk.^1^, ^2^, ^3^

For objective seizure documentation, in‐hospital monitoring using video‐EEG surveillance is considered the gold standard when a discrepancy between patient‐based documentation and actual seizure load is suspected. The duration of such in‐patient video‐EEG monitoring is mostly limited to a period of 1‐2 weeks, which again may lead to an undersampling of seizures^4^, ^5^ and may not represent the situation under real life conditions. That has justified the need to use ultralong‐term monitoring periods of months in order to establish a realistic view on seizure occurrence.^6^

We here report a clinical case in which ultralong‐term monitoring has been performed with a subcutaneously implanted EEG device^7^ to ascertain the real seizure frequency and the occurrence of seizure patterns in the right hemisphere in a patient with temporal lobe epilepsy who had an unknown seizure frequency and limited information on the predominant lateralization of seizure patterns. The information was relevant for the assessment of pharmacoresistance and to decide if the seizure load justifies the decision in favor of epilepsy surgery as an alternative treatment. Since the predominant seizure lateralization could only be partly established during in‐patient monitoring, seizures arising from the right temporal lobe were analyzed as main contributors to seizure load that proposed the patient as a suitable candidate for possible surgical intervention. Furthermore, the patient became aware of her seizure load only when using the objective EEG‐based seizure documentation.

## 2 CASE PRESENTATION

A 52‐year‐old‐female right‐handed geriatric nurse presented with seizures occurring from the age of 32 years on. From the disease onset, focal seizures with impaired awareness were reported for which she frequently had a complete amnesia. Others reported staring, oral and manual automatisms, and unresponsiveness lasting for 2‐3 minutes. In the first years of disease, focal aware symptoms in the form of epigastric sensations at times had been initial seizure symptoms. Despite various therapeutic regimens with anti‐seizure medications (carbamazepine, lamotrigine, oxcarbazepine, and levetiracetam), persisting seizures were reported at monthly intervals. Due to lack of awareness during the seizures, an underreporting was suspected early on after diagnosis.

Given the failure of multiple ASMs to control seizures, a first presurgical evaluation was performed in 2015. During 7 days of video‐EEG‐monitoring, eight seizures were recorded, six from sleep and two from wakefulness, none of them reported by the patient by pressing an alarm button. The first clinical sign on video‐EEG was an arousal or a slight change in behavior. The patient showed a gaze deviation to the right side, right manual automatisms and tonic posturing of the right limbs as well as unresponsiveness to stimuli. The EEG showed rhythmic activity 7‐8/s right temporal (Sp2, T2, T4) in all seizures about 20 seconds after clinical seizure onset. Epileptiform discharges appeared frequently over the right or left temporal region in approximately equal distribution.

A high‐resolution cerebral 3T MRI did not reveal an epileptogenic lesion. An FDG‐PET CT scan showed a small decrease in FDG uptake in the anterior right temporal lobe. Based on these findings, an invasive stereo EEG (SEEG)‐evaluation was recommended by the interdisciplinary case conference, yet rejected by the patient.

Following further medication changes to oxcarbazepine and levetiracetam and a withdrawal of her driving license after a traffic accident considered to be seizure‐related, she again presented in our outpatient clinic to obtain a driving allowance. A second video‐EEG monitoring was performed to verify the reported seizure freedom, yet it revealed two habitual seizures within 2 days on unchanged ongoing medication. The seizures again showed right temporal onset with rapid propagation to the left temporal region.

Despite up‐titration of her medication up to adverse effects, no complete seizure control was achieved, the patient continued to report up to three seizures from wakefulness per month. In another non‐invasive presurgical evaluation, six seizures with right temporal seizure onset were recorded, none of which with a patient alarm. Neuropsychological assessment revealed frontal, temporal, and parietal dysfunction with emphasis on the non‐dominant (right) hemisphere. In a subsequent invasive video‐EEG monitoring with nine depth EEG electrodes (six electrodes right temporal, three electrodes left temporal) over 9 days, a total of nine focal aware and unaware epileptic seizures occurred, one of them with transition to a bilateral tonic‐clonic seizure; only three of nine seizures were reported by a patient alarm. Intracranially, six seizures orginated in the right amygdala and three seizures in the left amygdala seizure onset. Semiology did not provide lateralizing information in eight of nine seizures, the only lateralizing feature was a sign of four with left‐sided arm extension during transition to a bilateral tonic‐clonic seizure.

Given a bilateral epileptogenesis, yet a ratio of 22:3 seizures suggesting a predominant right temporal onset, somewhat reduced chances for seizure freedom following epilepsy surgery were communicated to the patient. She did not agree to a surgical intervention. With continued antiseizure medication, she initially reported no seizures for 11 months until another unexplained fall down the stairs occurred and resulted in a coccygeal fracture. At this point, she was offered implantation of a system for ultralong EEG registration (UNEEG®) for a further evaluation of seizure frequency and right temporal involvement. The lead of the subcutaneous EEG device with three EEG channels was implanted over the right temporal lobe of the patient. The data were recorded at 207 Hz and bandpass filtered 0.5‐48 Hz equiripple FIR filter with a sidelobe attenuation of 40 dB and passband ripple of <0.1 dB.

**FIGURE 1:**
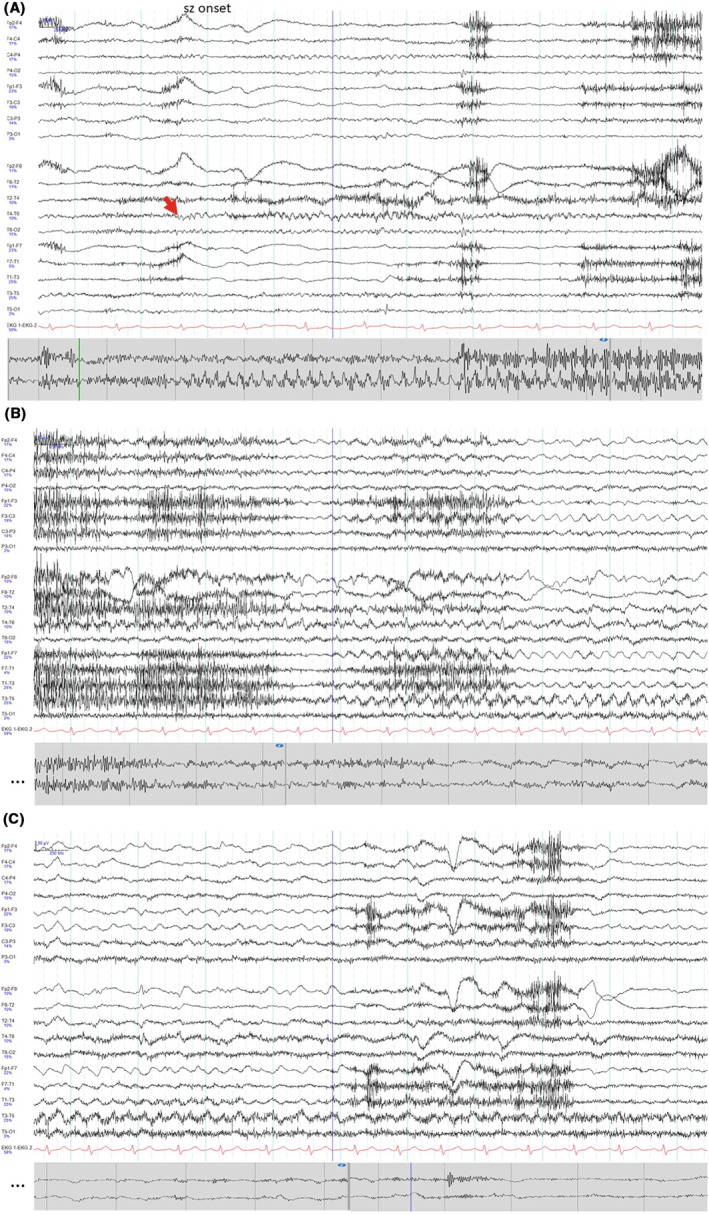
Simultaneous registration of a seizure in surface EEG (top) and subcutaneous EEG (bottom) at seizure onset (A, red arrow), during seizure evolution (B) and at seizure end (C) on the right temporal lobe. Both the initial rhythmic temporal theta pattern and the late rhythmic delta pattern are clearly visible, intermediate parts are obscured by muscle artifacts (B) in subcutaneous recordings, and the propagation to the left temporal lobe is visible only with the bilateral scalp recordings.

Simultaneous subcutaneous and scalp EEG recordings showed a high signal quality of subcutaneous ictal patterns (Figure 1).

During an outpatient monitoring of the initial 7 months, subcutaneous EEG showed 52 right temporal seizure patterns in contrast to two seizures documented in the patient diary which had occurred at daytime and was noted by her partner; 33 (63%) seizures occurred during nighttime (10 pm‐6 am).

**FIGURE 2:**
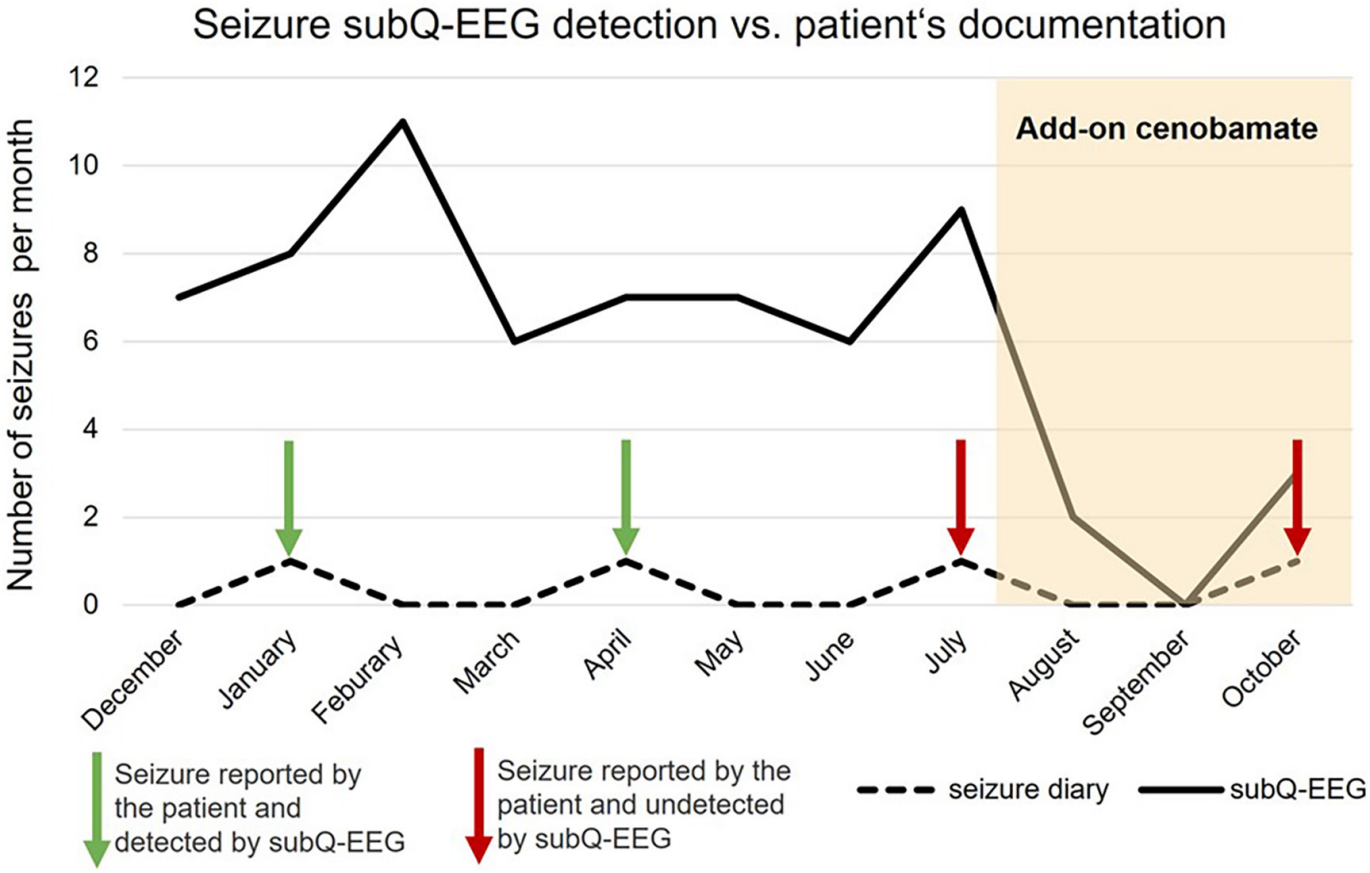
Comparison of the number of seizures documented by the patient and right temporal seizure patterns detected by subcutaneous EEG (subQ‐EEG) monitoring.

Following a new fall with a severe seizure‐related injury, cenobamate was added to her ASM at a maximal dosage of 150 mg/d, resulting in a decrease in seizure frequency as shown by subcutaneous EEG monitoring (Figure 2), yet with re‐occurrence of higher seizure rates after a “honeymoon period” and another fall with injury.

During a total recording period of 11 months, 66 right temporal seizure patterns were documented in subcutaneous EEG recordings, which was in striking contrast to only four seizures reported by the patient herself. Two of these seizures showed a timely coincidence with those detected in the subcutaneous EEG, whereas for the two other seizures, no electrographic ictal pattern was detected on the days reported in the patient's seizure diary.

Given the information provided by the ultralong‐term ambulatory subcutaneous EEG recordings, the patient now opted for a two‐thirds anterior resection of the right temporal lobe, given the high numbers of seizures with a right temporal ictal EEG patterns for which she was amnestic, and the severity of seizures.

## 3 DISCUSSION

This case shows that subcutaneous ultralong‐term EEG monitoring can provide relevant additional information beyond what patients can report on their seizures. Subcutaneous EEG provided high‐quality EEG recordings over a period of 11 months, confirming earlier reports on long‐term stability of recordings,^8^ on its good tolerability and safety,^9^ and allowing to clearly identify ictal electrographic patterns and to base seizure counting thereon. Added value of the diagnostic procedure led to the identification of a more than 10‐fold higher seizure frequency than reported by the patient herself in her seizure diary, thus confirming a gross underreporting of seizures as had been suggested by only 2 of 25 seizures reported also during in‐hospital monitoring. This led to a more valid judgment on the severity of epilepsy in this patient, and added evidence for insufficient efficacy of pharmacotherapy. Overall, this supported the decision in favor of alternative, non‐pharmacological treatment approaches. This can be epilepsy surgery^10^ or neuromodulatory treatment approaches (eg, VNS,^11^ DBS,^12^ RNS,^13^ or FCS^14^).

Moreover, device implantation over the temporal lobe in a patient considered a possible candidate for a surgical intervention provided additional evidence on the involvement of the right temporal lobe in focal seizures. The total number of electrographic seizure patterns recorded over the right temporal lobe was far higher than during three in‐patient video‐EEG monitoring (66 vs 25 Seizures), thus strongly suggesting an at least predominant role of this temporal lobe in seizure generation. Short durations of in‐patient monitoring have been shown to considerably undersample seizures and may provide insufficient information on the lateralization of seizure origin.^15^, ^16^ A correct lateralization of at least the predominant side of seizure generation is, however, a prerequisite for resective epilepsy surgery, laser‐based thermocoagulation^17^ or epicranial Focal Cortex Stimulation.^14^ Whereas the unilateral implantation used here could be based on prior imaging and EEG findings, other patients in whom lateralization of seizures is more unclear may particularly profit from future devices for ultralong‐term monitoring presently under investigation.^15^, ^18^ The case here shows that in patients in whom additional converging evidence is needed, a unilateral implantation may already provide relevant information which can contribute to decisions in favor of a focal intervention, as offered here in the form of a two‐thirds anterior temporal lobe resection.

In two seizures reported only by the patient, inaccuracy in seizure reporting cannot be ruled out as a reason for the discrepancy with subcutaneous recordings. There is thus a limitation in the interpretation of these two seizures that it is not possible to exclude that EEG‐based seizure detection was not sensitive enough or that the right temporal lobe was not affected in these cases. As muscle activity occurs not infrequently in temporal lobe seizures, particularly during oral automatisms, ictal EEG patterns may be obscured by this, suggesting that two‐channel EEG recordings may not always allow to detect more subtle ictal EEG patterns.

## AUTHOR CONTRIBUTIONS

MH and YN contributed to the acquisition and analysis of data; MH, AS‐B, and YN contributed to the drafting of the text and preparing of figures.

## CONFLICT OF INTEREST STATEMENT

MH has served as investigator in a clinical trial sponsored by UNEEG and recruited patients at the Freiburg Epilepsy Center. He has received honoraria for lectures from UNEEG. YN has served as investigator in a clinical trial sponsored by UNEEG and recruited patients at the Freiburg Epilepsy Center AS‐B has received institutional research support as PI in a clinical trial sponsored by UNEEG, and has received honoraria for lectures and advice from UNEEG. We confirm that we have read the Journal's position on issues involved in ethical publication and affirm that this report is consistent with those guidelines.

## INFORMED CONSENT

The patient gave written informed consent for publication of data.

## Data Availability

More detailed data will be made available upon reasonable request.

## References

[1] Mielke H , Meissner S , Wagner K , Joos A , Schulze‐Bonhage A . Which seizure elements do patients memorize? A comparison of history and seizure documentation. Epilepsia. 2020;61:1365–1375. 10.1111/epi.16550 32515852

[2] Schulze‐Bonhage A , Richardson MP , Brandt A , Zabler N , Dümpelmann M , San Antonio‐Arce V . Cyclical underreporting of seizures in patient‐based seizure documentation. Ann Clin Transl Neurol 10(10):1863–1872. 2023. 10.1002/acn3.51880. Epub ahead of print. 37608738 PMC10578895

[3] Serrand C , Rheims S , Faucanié M , Crespel A , Dinkelacker V , Szurhaj W , et al. Stratifying sudden death risk in adults with drug‐resistant focal epilepsy: the SUDEP‐CARE score. Eur J Neurol. 2023;30(1):22–31. 10.1111/ene.15566 36094672 PMC10087018

[4] Baud MO , Schindler K , Rao VR . Under‐sampling in epilepsy: limitations of conventional EEG. Clin Neurophysiol Pract. 2020;6:41–49. 10.1016/j.cnp.2020.12.002 33532669 PMC7829106

[5] Schulze‐Bonhage A , Bruno E , Brandt A , Shek A , Viana P , Heers M , et al. Diagnostic yield and limitations of in‐hospital documentation in patients with epilepsy. Epilepsia. 2022. 10.1111/epi.17307. Epub ahead of print. 35583131

[6] Gregg NM , Pal Attia T , Nasseri M , Joseph B , Karoly P , Cui J , et al. Seizure occurrence is linked to multiday cycles in diverse physiological signals. Epilepsia. 2023;64:1627–1639. 10.1111/epi.17607 37060170 PMC10733995

[7] Duun‐Henriksen J , Baud M , Richardson MP , Cook M , Kouvas G , Heasman JM , et al. A new era in electroencephalographic monitoring? Subscalp devices for ultra‐long‐term recordings. Epilepsia. 2020;61:1805–1817. 10.1111/epi.16630 32852091

[8] Viana PF , Remvig LS , Duun‐Henriksen J , Glasstetter M , Dümpelmann M , Nurse ES , et al. Signal quality and power spectrum analysis of remote ultra long‐term subcutaneous EEG. Epilepsia. 2021;62:1820–1828. 10.1111/epi.16969 34250608

[9] Weisdorf S , Duun‐Henriksen J , Kjeldsen M , Poulsen F , Gangstad S , Kjaer T . Ultra‐long‐term subcutaneous home monitoring of epilepsy‐490 days of EEG from nine patients. Epilepsia. 2019;60:2204–2214. 10.1111/epi.16360 31608435 PMC6899579

[10] Schulze‐Bonhage A , Zentner J . The preoperative evaluation and surgical treatment of epilepsy. Dtsch Arztebl Int. 2014;111:313–319. 10.3238/arztebl.2014.0313 24861650 PMC4038043

[11] Panebianco M , Rigby A , Marson AG . Vagus nerve stimulation for focal seizures. Cochrane Database Syst Rev. 2022;7:CD002896. 10.1002/14651858.CD002896.pub3 35833911 PMC9281624

[12] Fisher R , Salanova V , Witt T , Worth R , Henry T , Gross R , et al. Electrical stimulation of the anterior nucleus of thalamus for treatment of refractory epilepsy. Epilepsia. 2010;51:899–908. 10.1111/j.1528-1167.2010.02536.x 20331461

[13] Sun FT , Morrell MJ . The RNS system: responsive cortical stimulation for the treatment of refractory partial epilepsy. Expert Rev Med Devices. 2014;11:563–572. 10.1586/17434440.2014.947274 25141960

[14] Schulze‐Bonhage A , Hirsch M , Knake S , Kaufmann E , Kegele J , Rademacher M , et al. Focal cortex stimulation with a novel implantable device and antiseizure outcomes in 2 prospective multicenter single‐arm trials. JAMA Neurol. 2023;80(6):588–596. 10.1001/jamaneurol.2023.0066 37010826 PMC10071400

[15] Chiang S , Fan JM , Rao VR . Bilateral temporal lobe epilepsy: how many seizures are required in chronic ambulatory electrocorticography to estimate the laterality ratio? Epilepsia. 2022;63:199–208. 10.1111/epi.17113 34723396 PMC9056258

[16] Hirsch LJ , Mirro EA , Salanova V , Witt TC , Drees CN , Brown MG , et al. Mesial temporal resection following long‐term ambulatory intracranial EEG monitoring with a direct brain‐responsive neurostimulation system. Epilepsia. 2020;61:408–420. 10.1111/epi.16442 32072621 PMC7154711

[17] Kang JY , Wu C , Tracy J , Lorenzo M , Evans J , Nei M , et al. Laser interstitial thermal therapy for medically intractable mesial temporal lobe epilepsy. Epilepsia. 2016;57:325–334. 10.1111/epi.13284 26697969

[18] Stirling RE , Maturana MI , Karoly PJ , Nurse ES , McCutcheon K , Grayden DB , et al. Seizure forecasting using a novel sub‐scalp ultra‐long term EEG monitoring system. Front Neurol. 2021;12:713794. 10.3389/fneur.2021.713794 34497578 PMC8419461

